# Chromosome-level genome assembly of predatory *Arma chinensis*

**DOI:** 10.1038/s41597-024-03837-5

**Published:** 2024-09-04

**Authors:** Luyao Fu, Changjin Lin, Wenyan Xu, Hongmei Cheng, Dianyu Liu, Le Ma, Zhihan Su, Xiaoyu Yan, Xiaolin Dong, Chenxi Liu

**Affiliations:** 1 Sino-American Biological Control Laboratory, Institute of Plant Protection, Chinese Academy of Agricultural Sciences, Beijing, 100193 China; 2 College of Agriculture, Yangtze University, No. 1 Nanhuan Road, Jingzhou, 434025 Hubei China

**Keywords:** Genome, Entomology

## Abstract

*Arma chinensis* is a natural enemy that preys on various species and can suppress agricultural and forest pests in the orders Lepidoptera and Coleoptera. Here, we aimed to determine the genome of *A. chinensis* assembled at the chromosome-level using PacBio and Hi-C technologies. The assembled genome was 986 Mb, with a contig N50 of 2.40 Mb, scaffold N50 of 134.98 Mb, and BUSCO completeness of 96.10%. Hi-C data aided in anchoring the assembly onto seven chromosomes. A sequence of ~ 496.2 Mb was annotated as a repeat element, constituting 51.15% of the genome. We functionally annotated 84.79% of 20,853 predicted protein-encoding genes. This high-quality *A. chinensis* genome provides a novel genomic resource for future research on Pentatomidae insects.

## Background & Summary

**Fig. 1:**
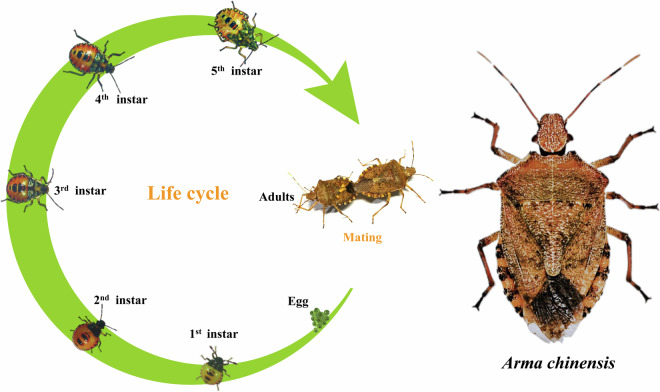
Seven stages of *Arma chinensis* life cycle. Eggs proceed through five nymphal instar stages, with final differentiation into adult males and females.

*Arma chinensis* is a true bug that belongs to the suborder Heteroptera and family Pentatomidae, encompassing all stink bugs. It is distributed primarily in China, Mongolia, the Korean Peninsula, Japan, and other East Asian regions^1^. Hemimetabolous *A. chinensis* has three major developmental life stages (egg, nymph, and adult), with the nymphal stage divided into five instars (Fig. 1). *Arma chinensis* preys on many species and can suppress agricultural and forest pests in the orders Lepidoptera and Coleoptera^1,2^. Like most terrestrial predatory arthropods, *A. chinensis* uses extraoral digestion for relatively large prey and obtains prey and nutrient concentrations through refluxing and non-refluxing while injecting hydrolytic enzymes^3–5^. The utilization of nutrition from its prey or artificial diets has been evaluated using bioassays^6,7^, nutrigenomics^8,9^ and metabolomics^10^. The chemoreception^11,12^ and aggregation-sex pheromones^13^ of *A. chinensis* have been functionally characterized and verified. Besides, *A. chinensis* has high tolerance of heat^14^, starvation^15^ and drought^16^, revealing ecophysiological adaptation to extreme environmental conditions. In addition, it has more tolerance to insecticidal pyrethroids than its prey^17^, suggesting that it has potential compatibility with chemical insecticides in pest management programs. Although biological control applications and the physiological characteristics of *A. chinensis* have been extensively studied, the lack of genome data has hindered knowledge of deeper gene functions in this species. Therefore, a high-quality genome of this species is needed to facilitate further exploration of the genetic and molecular mechanisms of Pentatomidae insects.

Herein, we constructed a high-quality chromosome-level reference genome for *A. chinensis* using PacBio long-read sequencing and Hi-C sequencing. The assembled genome is 986 Mb, with a contig N50 of 2.40 Mb, scaffold N50 of 134.98 Mb. The Hi-C sequences were further clustered and ordered into seven chromosomes. A sequence of ~496.2 Mb was annotated as a repeat element, constituting 51.15% of the genome. We predicted 20,853 protein-coding genes, of which 84.79% were functionally annotated. We also sequenced the developmental transcriptome of *A. chinensis*. This *A. chinensis* genome provides a novel genomic resource for future research on Pentatomidae insects.

## Methods

### Insect rearing and sample collection

*Arma chinensis* individuals were collected from a population reared in our laboratory in Beijing, China, for > 60 generations. The insects were fed with *Antheraea pernyi* pupae and reared at 26  ± 1 °C under 60 ± 5% relative humidity, and 14-h light: 10-h dark photoperiods. We sequenced the genome of female progeny that had been successively inbred for nine generations to reduce background noise. The surfaces of the insects were cleaned with 75% ethanol. Gut contents were removed to eliminate pollutants, then the specimens were stored in liquid nitrogen.

### Nucleic acid extraction and sequencing

Genomic DNA was extracted from *A. chinensis* tissues, using DNeasy Blood & Tissue Kits (QIAGEN, Hilden, Germany). The integrity of DNA was determined using an Agilent 4200 Bioanalyzer (Agilent Technologies, Palo Alto, CA, USA). Genomic DNA (8 μg) were sheared using g-Tubes (Covaris, Woburn, MA, USA) and concentrated with AMPure PB magnetic beads (Beckman Coulter, Brea, CA, USA). We constructed libraries using the Single Molecule Real Time (SMRT) bell template prep Kit 2.0 (Pacific Biosciences, Menlo Park, CA, USA). The libraries were size-selected on a BluePippin™ system (Sage Science, Inc., Beverly, MA, USA) with a ≥ 15 kb cutoff, followed by primer annealing and binding SMRT bell templates to polymerases with a DNA/Polymerase Binding Kit (Pacific Biosciences), then sequenced on a Sequel platform (Pacific Biosciences). A total of 11 SMRT cells were run. Size-selected SMRT bell libraries were prepared with a minimum fragment length of 10–20 kb. Medium- and large-insert libraries were sequenced using a PacBio Sequel system (Pacific Biosciences).

### Genome estimation and contig assembly

The genome was surveyed using a k-mer based method. The K-mer distribution was estimated using jellyfish (v2.2.10)^18^. The heterozygosity ratio was estimated using GenomeScope (v2.0)^19^. The size of the genome was calculated as: K-mer coverage/mean k-mer depth.

**Fig. 2:**
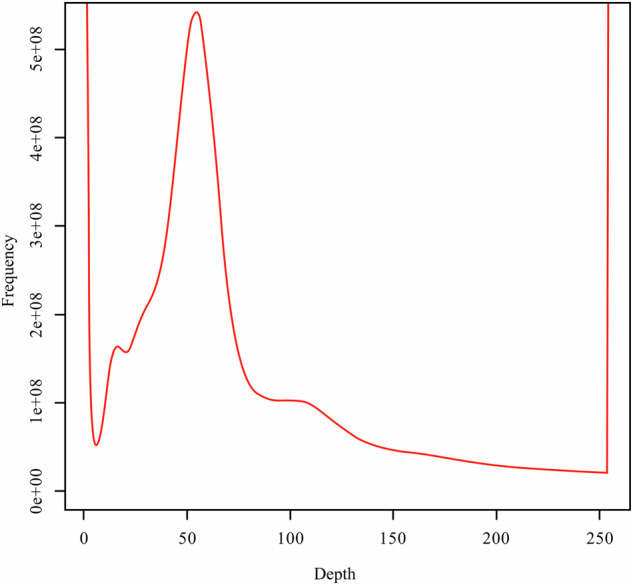
K-mer frequency distribution curve (k-mer = 17) of Illumina short reads of *A. chinensis* genome. X and Y axes respectively represent k-mer depth and k-mer frequency for a given depth.

The size of the *A. chinensis* genome estimated using the k-mer approach was ~826 Mb, with a heterozygosity of 1.01% and a repetitive sequence ratio of 32.25%, which suggests high heterozygosity and repetitive content (Fig. 2). We sequenced and assembled the genome of *A. chinensis* using SMRT (Pacific Biosciences)^20^ and Hi-C sequencing. We used 130 × coverage of SMRT sequences (128.0 Gb) for initial contig assembly, and SMRT sequences of 2.01 and 1.01 G, and contig N50 sizes of 0.20 and 0.87 Mb were respectively assembled using Canu (v2.0) and SmartDenovo (v1.0) (Supplementary Tables 1 and 2). Finally, we assembled the sequences using Quickmerge (v0.3), resulting in a 1.02 G sequence and a contig N50 size of 2.33 Mb (Supplementary Table 3). This assembly was slightly larger than the estimated genome size of 826 Mb. Considering that the size might be driven by underlying heterozygosity, we also reduced the assembly size to 986 Mb by scaffolding with Redundans. However, the Redundans assembly increased contig N50 to 2.40 Mb (Supplementary Table 4). We called blasr using smrtlink 5.0 with three generations of Subreads.bam, with the optional parameters: bam, bestn 5; minMatch, 18; nproc, 4; minSubread Length, 1,000; minAln Length 500; minPctSimilarity, 70; minPctAccuracy, 70; hitPolicy randombest, randomSeed and arrow correction was applied to the assembly result. Three generations of corrected genome sequences were obtained. Pilon v1.22 default parameters were combined with second-generation data for correction^21^.

### Karyotype analysis of *A. chinensis*

We analyzed the correctness of the *A. chinensis* genome assembly using Hi-C data. We fixed, stained, and counted the number of chromosomes. Briefly, the lateral margin of the abdominal side of adult males was cut, then they were immersed in water for 30 min, fixed for 14 h in methyl alcohol- acetic acid (3:1 v/v) and stored in 70% alcohol at 4 °C. The gonads were dissected in 70% ethanol and crushed in a drop of 45% acetic acid. The coverslips were removed using dry ice as a decoverslipping agent^22^, then the slides were dehydrated in fresh fixative and air-dried.

We used the Feulgen–Giemsa method^23^ and an Olympus OV100microimaging system (Olympus, Tokyo, Japan) for standard karyotype analysis. Karyotypes and male meiosis were evaluated in *A. chinensis* based on slides prepared from male gonads (Supplementary Figure 1). Analyses of metaphase I (Supplementary Figure 1g), anaphase (Supplementary Figure 1h), and metaphase II (Supplementary Figure 1i) revealed that *A. chinensis* possessed a diploid chromosome set (2n = 14) comprising six autosomal pairs and two sex chromosomes.

### Chromosome-scale assembly of *A. chinensis*

Data processed by Illumina high-throughput sequencing was restored to the raw image format and transformed to sequenced reads with adapters and low-quality calling bases. We avoided alignment errors by filtering and trimming the raw reads to create clean reads. The Hi-C reads were aligned using Bowtie2 (v2.0.5) to orient the primary contigs along the chromosomes^24^. Clean reads were first aligned to the reference genome using the bowtie2 end-to-end algorithm. Unmapped reads primarily comprised chimeric fragments spanning ligation junctions. HiC-Pro (v2.7.8) detected the ligation site using an exact matching procedure and aligned five fractions of the read on the genome^25^. Both mapping steps were merged into a single alignment file. Low-mapping-quality reads, multiple hits, and singletons were filtered out. Duplicates were removed, and reads that were uniquely mapped to the reference genome were retained. Clustering, ordering, and orientation proceeded using The LACHESIS assembly package (https://github.com/shendurelab/LACHESIS)^26^. Based on the agglomerative hierarchical clustering algorithm, scaffolds were clustered into N groups. The longest acyclic spanning tree (“trunk”) was built based on relationships between the normalized Hi-C interactions. Scaffolds excluded from the trunk were reinserted at sites that maximized the linkages between adjacent scaffolds. For each chromosomal cluster, we obtained the exact scaffold order of the internal groups and traversed all directions of the scaffolds using a weighted directed acyclic graph to predict the orientation of each scaffold.

**Fig. 3:**
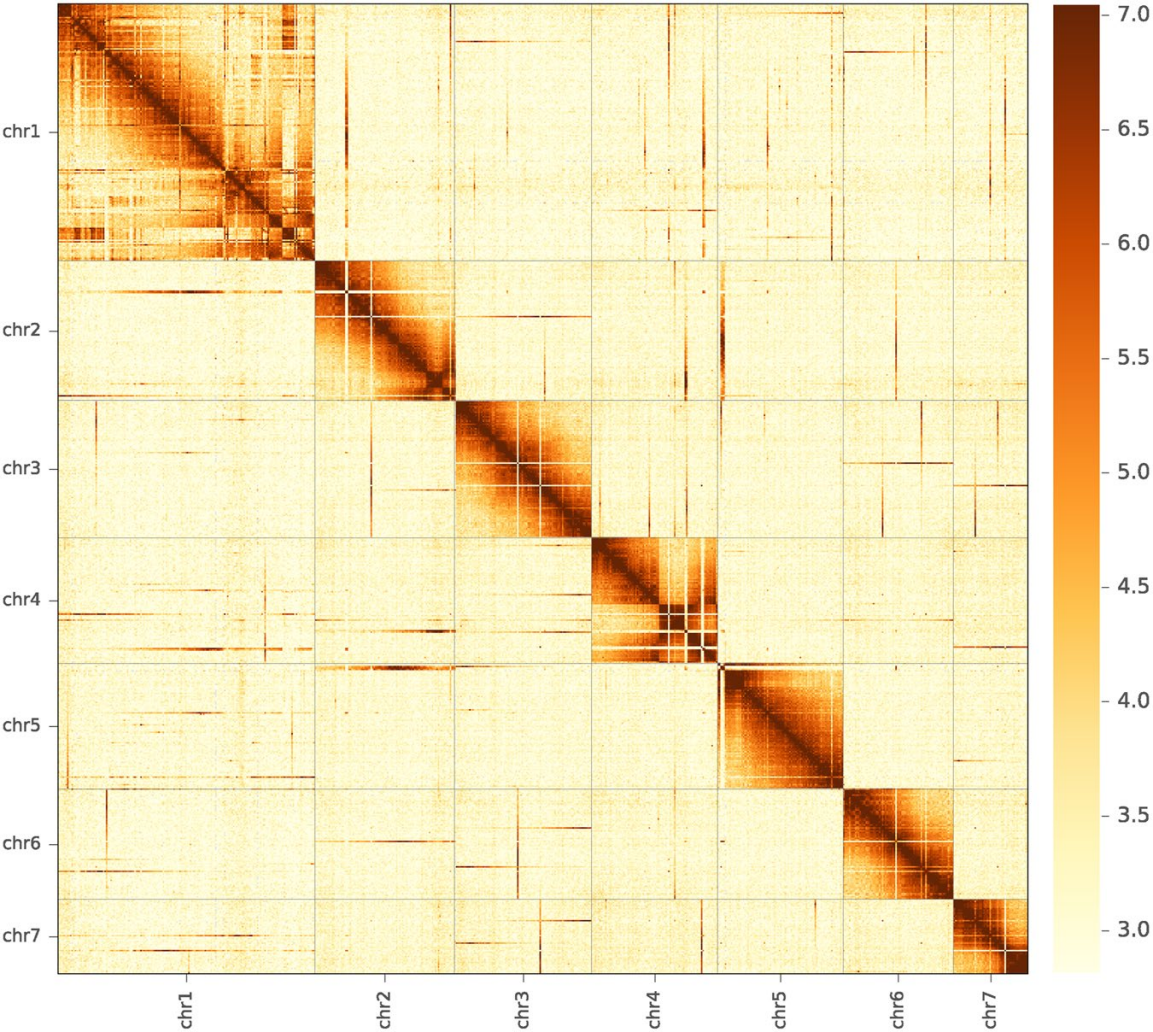
Hi-C interaction map of assembled *A. chinensis* scaffolds. Darker colors indicate a higher frequency of chromatin interaction. Clear separation of chromosome boundaries and limited off-diagonal interactions are visible, supporting the global structure of chromosome-scale scaffolds.

**Fig. 4:**
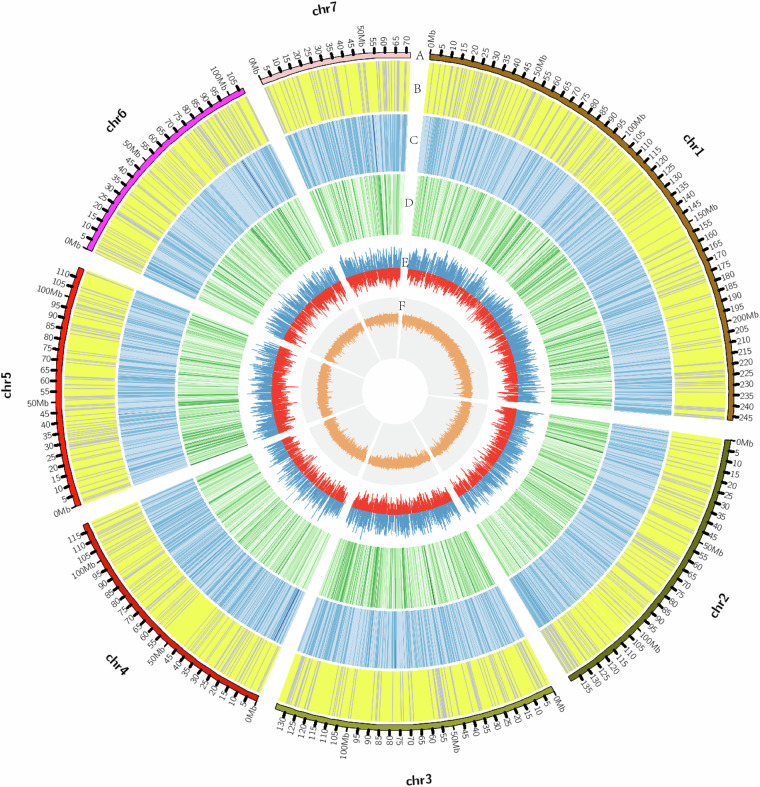
Circos plot of *A. chinensis* genome profile. (**A**) Chromosome number and length. (**B**) Non-coding RNAs: yellow, tRNA; purple, other ncRNAs. (**C**) Abundance of repetitive sequences. Dark blue indicates greater quantity. (**D**) Abundance of genes. Dark green indicates greater quantity. (**E**) Transcriptome gene expression calculated by log2 FPKM. Red and blue, upregulated and downregulated expression, respectively. (**F**) GC content (10 k used as calculating unit).

Starting with the draft assembly, Hi-C data were used to correct mis-joins, scaffolds, and merge overlaps, generating an assembled *A. chinensis* genome with chromosome-length scaffolds. Finally, 1,357 contigs/620 scaffolds (97.70%) were clustered into seven groups (Figs. 3 and 4), that were consistent with previous karyotype analyses of *A. chinensis*. The 1,357 clustered contigs corresponded to a length of 967.93 Mb (99.77% of the length of the corrected contigs [970.20 Mb] and 97.7% of the total number of contigs [1,389] according to LACHESIS; Supplementary Tables 5 and 6). These results showed that the assembled *A. chinensis* draft genome has a high level of continuity and completeness.

### Repeat identification and non-coding RNA annotation

We used homologous sequence and *de novo* repeat identification to annotate repeat elements in *A. chinensis*. First, RepeatMasker (v4.09) and RepeatProteinMask (v4.09) identified tandem and interspersed repeats according to their sequence similarity with the repeats deposited in RepBase (http://www.girinst.org/repbase/)^27^. Subsequently, RepeatModeler (open-1.0.11) trained a repeat database using the NCBI blast approach (-engine NCBI), and the repeat elements were annotated according to the database built using RepeatMasker (v4.09). Tandem repeats were also predicted and annotated directly using TRF software built into RepeatMasker (v4.09). Finally, we identified a ~496.2 Mb sequence as a repeat element in *A. chinensis*, constituting ~51.15% of the genome (Supplementary Table 7). We found that DNA transposons accounted for 5.08% of the genome, whereas long interspersed nuclear elements (LINEs) and long terminal repeat (LTR) retrotransposons accounted for 17.09% and 4.83% of the genome, respectively. LINEs constituted most of the repeat elements in *A. chinensis*.

We predicted tRNAs using tRNAscan-SE65 (http://lowelab.ucsc.edu/tRNAscan-SE/)^28^. Other non-coding RNAs, such as rRNAs, snRNAs, and miRNAs, were identified by homologous searches for sequences deposited in the Rfam database (http://rfam.xfam.org/)^29^. All parameters were set to their default values. Different quantities of non-protein-coding miRNAs, including tRNA, rRNA, and snRNA genes were predicted, comprising 0.2914% of the genome (Supplementary Table 8).

### Gene prediction and functional annotation

We combined *ab initio*-, RNA-seq-, and protein homology-based approaches to predict protein-coding genes in *A. chinensis*. For protein homology-based predictions, we downloaded the protein sequences of *Acyrthosiphon pisum*, *Cimex lectularius*, *Halyomorpha halys*, and *Oncopeltus fasciatus* and aligned them to the assembled scaffolds using TBLASTN (e-value < 1e-5), which is a mode of operation for BLAST that aligns protein sequences to a nucleotide database translated in all six frames. Alignments within 20 kb were merged, and those with coverage > 85% and identity > 75% were retained. Gene models were annotated according to their alignment using Genewise (v2.2.0)^30^ and we applied Augustus (v3.3)^31^, SNAP (v11-29-2013)^32^, Glimmer HMM (v 3.0.2)^33^, and GeneMark-ES/ET/EP (v4.68)^34^ for *de novo* predictions. We generated RNA-seq data from different developmental stages of *A. chinensis* and aligned them to the scaffolds using Program to Assemble Spliced Alignment (PASA) (v2.4.1)^35^ and TopHat (v 2.0.9)^36^ for RNA-seq-based prediction. All results were finally integrated into a single high-confidence gene model set using EVidenceModeler (v1.1.1)^37^. We predicted 20,853 genes with an average sequence length of 14,007 bp, an average coding sequence length of 1,144 bp, and five exons per gene. The average sequence lengths of the exons and introns were 199 and 2,940 bp, respectively (Supplementary Table 9). A comparison of closely related species revealed more genes, suggesting that our gene annotation was complete (Supplementary Table 10). Supplementary Figure 2 shows the distribution of the mRNA, CDS, exon, and intron lengths between *A. chinensis* and closely related species. Supplementary Figure 3 shows the distribution of exon numbers between *A. chinensis* and closely related species.

We aligned the predicted protein-coding gene sequences with public functional databases for protein-coding gene functional annotation using BLASTX/BLASTP with a threshold E-value of 1e-5, including SwissPro (https://web.expasy.org/docs/swiss-prot_guideline.htm), NCBI nucleotide sequence database (NT) (https://www.ncbi.nlm.nih.gov/nucleotide/), NCBI non-redundant databases (NR) (ftp://ftp.ncbi.nlm.nih.gov/blast/db/FASTA/nr.gz), Pfam (http://xfam.org/), EggNOG (http://eggnogdb.embl.de/), Gene Ontology (GO) (http://geneontology.org/page/go-database), and the Kyoto Encyclopedia of Genes and Genomes (KEGG; http://www.genome.jp/kegg/). We functionally annotated 17,681 genes that accounted for 84.79% of all unigenes (Supplementary Table 11).

### Transcriptome sequencing

We collected fresh samples of eggs, mixed first-, second-, third-, and fourth-instar nymphs, mixed female, and mixed male adults (*n = *24 samples; *n = *3 biological repeats/developmental stage). Total RNA was extracted using TRIzol reagent as described by the manufacturer (Invitrogen, Carlsbad, CA, USA). The integrity of the total RNA was determined by 1% agarose gel electrophoresis, and total RNA was quantified using 2100 RNA Nano 6000 Assay Kits (Agilent Technologies, Santa Clara, CA, USA). We prepared RNA-Seq libraries using TruSeq RNA sample preparation kits (Illumina, San Diego CA, USA) and sequenced them using an HiSeq PE150 platform (Illumina). Raw RNA-seq reads were processed to remove adapters and low-quality sequences using SeqTk (https://github.com/lh3/seqtk). Cleaned reads were used to generate a *de novo* RNA-seq assembly using the Trinity program with default parameters^38^. The resulting reads were processed *via* genome mapping using Hisat2 (version:2.0.4)^39^ against the *A. chinensis* genome.

## Data Records

Pacific Biosciences, Illumina, and Hi-C sequencing data were deposited in the NCBI GenBank under accession number JAGJRN000000000^40^. Developmental transcriptome data for eggs, larvae, and adults were deposited in the NCBI Sequence Read Archive under accession number PRJNA1123459^41^.

## Technical Validation

### Genome assembly assessment

We analyzed the genome assembly to benchmark sets of universal single-copy orthologs (BUSCOs) to assess the completeness of the assembly. The *A. chinensis* gene set and genome had 96.1% complete (C), and 2.8% missing (M) BUSCOs (Supplementary Table 12). The distribution of GC-depth indicated that the assembled *A. chinensis* genome did not contain any visible bacterial contamination (Supplementary Figure 4). Therefore, we concluded that the *A. chinensis* dataset was comprehensive enough for further downstream analysis.

### Chromosomal clustering assessment

The basic principles of HiC analysis are that intra-chromosome contacts are stronger than inter-chromosome contacts and that interactions weaken as distance increases. Consequently, interactions near the diagonal line are stronger than those located further from the diagonal line, and close bins are closely related in the heatmap. We separated the chromosomes predicted by LACHESIS into bins of equal lengths of 1 Mb or 500 kb, and constructed a heat map based on interaction signals revealed by valid mapped read pairs between bins. Failure of the heat map to conform to these rules suggested errors in the assembly results.

## Supplementary information

Chromosome-level genome assembly of predatory Arma chinensis

## Data Availability

No custom codes were used in this study. All bioinformatics tools and software applications were used according to their respective manuals and protocols. The specific software versions and parameters used are detailed in the Methods section.

## References

[1] Zou, D. *et al*. Taxonomic and bionomic notes on *Arma chinensis* (Fallou). *Zootaxa* **3382**, 41–53 (2012).

[2] Liu J., Liu X., Liao J., Li C. Biological performance of *Arma chinensis* on three preys *Antheraea pernyi*, *Plodia interpunctella* and *Leptinotarsa decemlineata*. *International Journal of Pest Management*, 1-8 (2023).

[3] Cohen, A. C. Extra-oral digestion in predaceous terrestrial arthropoda. *Annual Review of Entomology* **40**, 85–103 (1995).

[4] Cohen, A. C. Solid-to-Liquid feeding: the inside(s) story of extra-oral digestion in predaceous arthropoda. *American Entomologist* **44**, 103–117 (1998).

[5] Cantón, P. E. & Bonning, B. C. Extraoral digestion: outsourcing the role of the hemipteran midgut. *Current Opinion in Insect Science* **41**, 86–91 (2020). 32823203 10.1016/j.cois.2020.07.006

[6] Zou, D. Y. *et al*. A meridic diet for continuous rearing of *Arma chinensis* (Hemiptera: Pentatomidae: Asopinae). *Biological Control* **67**, 491–497 (2013).

[7] Zou, D. Y. *et al*. Performance and cost comparisons for continuous rearing of *Arma chinensis* (Hemiptera: Pentatomidae: Asopinae) on a zoophytogenous artificial diet and a secondary prey. *Journal of Economic Entomology* **108**, 454–461 (2015). 26470156 10.1093/jee/tov024

[8] Zou D. *et al*. Nutrigenomics in *Arma chinensis*: transcriptome analysis of *Arma chinensis* fed on artificial diet and Chinese oak silk moth *Antheraea pernyi* pupae. *PLoS ONE* **8** (2013). 10.1371/journal.pone.0060881 PMC3623872 23593338

[9] Zou D. *et al*. Differential proteomics analysis unraveled mechanisms of *Arma chinensis* responding to improved artificial diet. *Insects* **13** (2022). 10.3390/insects13070605 PMC9319121 35886781

[10] Guo, Y., Liu, C. X., Zhang, L. S., Wang, M. Q., Chen, H. Y. Sterol content in the artificial diet of *Mythimna separata* affects the metabolomics of *Arma chinensis* (Fallou) as determined by proton nuclear magnetic resonance spectroscopy. *Archives of Insect Biochemistry and Physiology* **96** (2017). 10.1002/arch.21426 29024237

[11] Wu S. *et al*. Analysis of chemosensory genes in full and hungry adults of *Arma chinensis* (Pentatomidae) through antennal transcriptome. *Frontiers in Physiology* **11** (2020). 10.3389/fphys.2020.588291 PMC7677363 33240109

[12] Wang Z. *et al*. Genome-wide analysis of gustatory receptor genes and identification of the fructose gustatory receptor in *Arma chinensis*. *Heliyon* **10** (2024). 10.1016/j.heliyon.2024.e30795 PMC11096949 38765039

[13] Wu, H. *et al*. Identification and field verification of aggregation-sex pheromone from the predaceous bug, *Arma chinensis*. *Chemoecology* **29**, 235–245 (2019).

[14] Meng, J.-Y., Yang, C.-L., Wang, H.-C., Cao, Y. & Zhang, C.-Y. Molecular characterization of six heat shock protein 70 genes from *Arma chinensis* and their expression patterns in response to temperature stress. *Cell Stress and Chaperones* **27**, 659–671 (2022). 36264419 10.1007/s12192-022-01303-y PMC9672165

[15] Pan, M., Zhang, H., Zhang, L. & Chen, H. Effects of starvation and prey availability on predation and dispersal of an omnivorous predator *Arma chinensis* Fallou. *Journal of Insect Behavior* **32**, 134–144 (2019).

[16] Liu, J., Liao, J. & Li, C. Bottom‐up effects of drought on the growth and development of potato, *Leptinotarsa decemlineata* Say and *Arma chinensis* Fallou. *Pest Management Science* **78**, 4353–4360 (2022). 35775398 10.1002/ps.7054

[17] Wang Z. *et al*. Detoxification and neurotransmitter clearance drive the recovery of *Arma chinensis* from β-cypermethrin-triggered knockdown. *Journal of Hazardous Materials* **476** (2024). 10.1016/j.jhazmat.2024.135175 39002489

[18] Marçais, G. & Kingsford, C. A fast, lock-free approach for efficient parallel counting of occurrences of k-mers. *Bioinformatics* **27**, 764–770 (2011). 21217122 10.1093/bioinformatics/btr011 PMC3051319

[19] Ranallo-Benavidez T. R., Jaron K. S., Schatz M. C. GenomeScope 2.0 and Smudgeplot for reference-free profiling of polyploid genomes. *Nature Communications* **11** (2020). 10.1038/s41467-020-14998-3 PMC7080791 32188846

[20] Hackl, T., Hedrich, R., Schultz, J. & Förster, F. proovread: large-scale high-accuracy PacBio correction through iterative short read consensus. *Bioinformatics* **30**, 3004–3011 (2014). 25015988 10.1093/bioinformatics/btu392 PMC4609002

[21] Wang J. *et al*. Pilon: an integrated tool for comprehensive microbial variant detection and genome assembly improvement. *PLoS ONE* **9** (2014). 10.1371/journal.pone.0112963 PMC4237348 25409509

[22] Sabarinath, B., Protyusha, G. B., Sivapathasundharam, B. & Dhanarathna, S. Role of dry ice in decoverslipping of microscopic slides: A new insight. *Journal of Oral and Maxillofacial Pathology* **27**, 598–602 (2023). 38033942 10.4103/jomfp.jomfp_332_22 PMC10683887

[23] Grozeva, S. & Nokkala, S. Chromosomes and their meiotic behavior in two families of the primitive infraorder dipsocoromorpha (Heteroptera). *Hereditas* **125**, 31–36 (2004).

[24] Langmead, B. & Salzberg, S. L. Fast gapped-read alignment with Bowtie 2. *Nature Methods* **9**, 357–359 (2012). 22388286 10.1038/nmeth.1923 PMC3322381

[25] Servant N. *et al*. HiC-Pro: an optimized and flexible pipeline for Hi-C data processing. *Genome Biology* **16** (2015). 10.1186/s13059-015-0831-x PMC4665391 26619908

[26] Burton, J. N. *et al*. Chromosome-scale scaffolding of *de novo* genome assemblies based on chromatin interactions. *Nature Biotechnology* **31**, 1119–1125 (2013). 24185095 10.1038/nbt.2727 PMC4117202

[27] Jurka, J. *et al*. Repbase Update, a database of eukaryotic repetitive elements. *Cytogenetic and Genome Research* **110**, 462–467 (2005). 16093699 10.1159/000084979

[28] Lowe, T. M. & Eddy, S. R. tRNAscan-SE: a program for improved detection of transfer RNA genes in genomic sequence. *Nucleic Acids Research* **25**, 955–964 (1997). 9023104 10.1093/nar/25.5.955 PMC146525

[29] Griffiths-Jones, S. Rfam: annotating non-coding RNAs in complete genomes. *Nucleic Acids Research* **33**, D121–D124 (2004). 10.1093/nar/gki081 PMC540035 15608160

[30] Birney, E., Clamp, M. & Durbin, R. GeneWise and Genomewise. *Genome Research* **14**, 988–995 (2004). 15123596 10.1101/gr.1865504 PMC479130

[31] Stanke, M., Steinkamp, R., Waack, S. & Morgenstern, B. AUGUSTUS: a web server for gene finding in eukaryotes. *Nucleic Acids Research* **32**, W309–W312 (2004). 15215400 10.1093/nar/gkh379 PMC441517

[32] Korf I. Gene finding in novel genomes. **5**, 1-9 (2004). 10.1186/1471-2105-5-59 PMC421630 15144565

[33] Majoros, W. H., Pertea, M. & Salzberg, S. L. TigrScan and GlimmerHMM: two open source ab initio eukaryotic gene-finders. *Bioinformatics* **20**, 2878–2879 (2004). 15145805 10.1093/bioinformatics/bth315

[34] Brůna T., Lomsadze A., Borodovsky M. GeneMark-EP+: eukaryotic gene prediction with self-training in the space of genes and proteins. *NAR Genomics and Bioinformatics* **2** (2020). 10.1093/nargab/lqaa026 PMC7222226 32440658

[35] Haas, B. J. Improving the *Arabidopsis* genome annotation using maximal transcript alignment assemblies. *Nucleic Acids Research* **31**, 5654–5666 (2003). 14500829 10.1093/nar/gkg770 PMC206470

[36] Kim D., Salzberg S. L. TopHat-Fusion: an algorithm for discovery of novel fusion transcripts. *Genome Biology* **12** (2011). 10.1186/gb-2011-12-8-r72 PMC3245612 21835007

[37] Haas B. J. *et al*. Automated eukaryotic gene structure annotation using EVidenceModeler and the Program to Assemble Spliced Alignments. *Genome Biology* **9** (2008). 10.1186/gb-2008-9-1-r7 PMC2395244 18190707

[38] Garber, M., Grabherr, M. G., Guttman, M. & Trapnell, C. Computational methods for transcriptome annotation and quantification using RNA-seq. *Nature Methods* **8**, 469–477 (2011). 21623353 10.1038/nmeth.1613

[39] Kim, D., Langmead, B. & Salzberg, S. L. HISAT: a fast spliced aligner with low memory requirements. *Nature Methods* **12**, 357–360 (2015). 25751142 10.1038/nmeth.3317 PMC4655817

[40] *NCBI GenBank*. https://identifiers.org/ncbi/insdc:JAGJRN000000000 (2024).

[41] *NCBI Sequence Read Archive*. https://identifiers.org/ncbi/insdc.sra:SRP513644 (2024).

