# Publisher Correction: Chromosome-level genome assembly of predatory *Arma chinensis*

**DOI:** 10.1038/s41597-024-04069-3

**Published:** 2024-11-15

**Authors:** Luyao Fu, Changjin Lin, Wenyan Xu, Hongmei Cheng, Dianyu Liu, Le Ma, Zhihan Su, Xiaoyu Yan, Xiaolin Dong, Chenxi Liu

**Affiliations:** 1 Sino-American Biological Control Laboratory, Institute of Plant Protection, Chinese Academy of Agricultural Sciences, Beijing, 100193 China; 2 College of Agriculture, Yangtze University, No. 1 Nanhuan Road, Jingzhou, 434025 Hubei China

**Keywords:** Genome, Entomology, Genome, Entomology

Correction to: *Scientific Data* 10.1038/s41597-024-03837-5, published online 04 September 2024

The PDF version of this originally published article was the uncorrected proof; it has now been replaced by the corrected version. The original article has been corrected.

