# Supplementary material for: Chromosome-level genome assembly of predatory Arma chinensis
Source: Sci Data. 2024 Sep 4;11:962. doi: 10.1038/s41597-024-03837-5 (PMC11374891; doi:10.1038/s41597-024-03837-5)
Supplement: Supplementary file 1 — Chromosome-level genome assembly of predatory Arma chinensis [file 41597_2024_3837_MOESM1_ESM.docx]

### **Supplementary Information**

**Chromosome-level genome assembly of predatory *Arma chinensis***

Luyao Fu, Changjin Lin, Wenyan Xu, Hongmei Cheng, Dianyu Liu, Le Ma, Zhihan Su, Xiaoyu Yan, Xiaolin Dong, Chenxi Liu

**Supplementary Table 1.** Assembly with Canu pipeline

| **Stat Type** | **Contig Length (bp)** | **Contig Number** |
| --- | --- | --- |
| Total | 2,008,219,811 | 25,398 |
| Max_length | 9,368,182 | - |
| Number ≥ 2 kb | - | 25,361 |
| N50 | 204,051 | 1,671 |
| N60 | 112,986 | 3,013 |
| N70 | 68,754 | 5,328 |
| N80 | 45,054 | 8,975 |
| N90 | 29,133 | 14,495 |

**Supplementary Table 2.** Assembly with smart-de novo pipeline

| **Stat Type** | **Contig Length (bp)** | **Contig Number** |
| --- | --- | --- |
| Total | 1,005,567,962 | 2,815 |
| Max_length | 5,692,529 | - |
| Number ≥ 2 kb | - | 2,815 |
| N50 | 871,459 | 315 |
| N60 | 690,649 | 445 |
| N70 | 505,569 | 616 |
| N80 | 352,534 | 853 |
| N90 | 180,530 | 1,247 |

**Supplementary Table 3.** Assembly with Quickmerge pipeline

| **Stat Type** | **Contig Length (bp)** | **Contig Number** |
| --- | --- | --- |
| Total | 1,017,430,925 | 1,449 |
| Max_length | 11,945,071 | - |
| Number ≥ 2 kb | - | 1,449 |
| N50 | 2,332,648 | 122 |
| N60 | 1,854,743 | 172 |
| N70 | 1,267,945 | 237 |
| N80 | 822,968 | 336 |
| N90 | 442,092 | 498 |

**Supplementary Table 4.** Scaffolding with Redundans

| **Stat Type** | **Contig Length (bp)** | **Contig Number** |
| --- | --- | --- |
| Total | 986,877,803 | 1,199 |
| Max_length | 11,945,071 | - |
| Number ≥ 2 kb | - | 1,199 |
| N50 | 2,403,708 | 116 |
| N60 | 1,917,430 | 162 |
| N70 | 1,387,659 | 222 |
| N80 | 910,595 | 311 |
| N90 | 520,885 | 453 |

**Supplementary Table 5.** Hi-C assembly

| **Item** | **Contig_len (bp)** | **Contig_num** | **Scaffold_len (bp)** | **Scaffold_num** |
| --- | --- | --- | --- | --- |
| Total | 970,197,563 | 1,389 | 970,274,463 | 620 |
| Max | 11,925,682 | - | 246,887,656 | - |
| Number ≥ 2000 bp | - | 1,389 | - | 620 |
| N50 | 2,160,000 | 128 | 134,981,943 | 3 |
| N60 | 1,599,440 | 181 | 118,614,055 | 4 |
| N70 | 1,100,000 | 253 | 114,631,289 | 5 |
| N80 | 762,642 | 360 | 107,050,919 | 6 |
| N90 | 400,000 | 531 | 71,898,614 | 7 |

**Supplementary Table 6.** Contig/Scaffold clustering

| **Item** | **Value** |
| --- | --- |
| Number of sequences in draft genome | 1,389 |
| Length of sequence in draft genome (bp) | 970,197,563 |
| Number of sequences in clustering | 1,357 |
| Rate of number in clustering (%) | 97.7 |
| Length of sequences in clustering (bp) | 967,932,628 |
| Rate of length in clustering (%) | 99.77 |

**Supplementary Table 7.** Repeat sequence identification

| **Type** | **Repeat Size** | **% of Genome** |
| --- | --- | --- |
| RepeatMasker | 29,620,771 | 3.05 |
| ProteinMask | 108,352,973 | 11.17 |
| Denovo | 478,238,408 | 49.29 |
| TRF | 21,235,220 | 2.19 |
| Total | 496,217,998 | 51.15 |

**Supplementary Table 8.** Non-protein-coding RNA prediction

| **Type** | | **Copy (w*)** | **Average Length (bp)** |  | **Total Length (bp)** | **% of Genome** |
| --- | --- | --- | --- | --- | --- | --- |
| miRNA | | 860 | 214.68140 |  | 184,626 | 0.01903 |
| tRNA | | 34,589 | 72.96554 |  | 2,523,805 | 0.26013 |
| rRNA | 18S | 161 | 94 |  | 15,173 | 0.001564 |
|  | 28S | 106 | 170 |  | 18,067 | 0.001862 |
|  | 5.8S | 9 | 93 |  | 838 | 0.000086 |
|  | 5S | 688 | 68 |  | 46,980 | 0.004842 |
| snRNA | CD-box | 36 | 178.88889 |  | 6440 | 0.00066 |
|  | HACA-box | 21 | 168.00000 |  | 3528 | 0.00036 |
|  | splicing | 239 | 116.90795 |  | 27,941 | 0.00288 |

**Supplementary Table 9.** *A. chinensis* annotation

| **Gene Set** | | **Gene**  **Number** | **Average Gene Length (bp)** | **Average CDS Length (bp)** | **Average Exons per Gene** | **Average Exon Length (bp)** | **Average Intron Length (bp)** |
| --- | --- | --- | --- | --- | --- | --- | --- |
| Denovo | Augustus | 34582 | 14,735.85 | 841.45 | 4.03 | 208.9 | 4589.79 |
|  | SNAP | 143,826 | 10,262.43 | 548.76 | 5.8 | 94.66 | 2025.94 |
|  | GlimmerHMM | 19,399 | 46,580.74 | 1574 | 12.36 | 127.3 | 3962.2 |
|  | GeneMark | 55,934 | 7901.73 | 768.62 | 4.65 | 165.2 | 1953.84 |
|  | *Acyrthosiphon pisum* | 15,203 | 2670.57 | 630.94 | 2.95 | 213.92 | 1047.29 |
| Homoloy | *Cimex lectularius* | 13,758 | 4448.71 | 866.16 | 4.22 | 205.07 | 1112.3 |
|  | *Halyomorpha halys* | 18,801 | 5053.15 | 909.51 | 4.41 | 206.04 | 1214.63 |
|  | *Oncopeltus fasciatus* | 17,824 | 2508.3 | 574.77 | 2.68 | 214.27 | 1150.22 |
| cDNA | | 33,028 | 33,350.45 | 1187.61 | 7.74 | 281.04 | 4626.06 |
| EVM | | 20,853 | 14,007.35 | 1076.07 | 5.40 | 199.30 | 2940.45 |

**Supplementary Table 10.** Closely related species annotation

| **Gene set** | **Gene**  **Number** | **Average Gene Length (bp)** | **Average CDS Length (bp)** | **Average Exons per Gene** | **Average Exon Length (bp)** | **Average Intron Length (bp)** |
| --- | --- | --- | --- | --- | --- | --- |
| *Halyomorpha halys* | 14,454 | 22,567.6 | 1445.21 | 7.39 | 195.44 | 3403.41 |
| *Acyrthosiphon pisum* | 18,616 | 8345.8 | 1346.55 | 6.04 | 222.84 | 1417.54 |
| *Cimex lectularius* | 11,936 | 19,239.73 | 1593.87 | 8.08 | 197.37 | 2501.49 |
| *Oncopeltus fasciatus* | 19,615 | 11,484.8 | 890.38 | 5.07 | 175.51 | 2602.07 |

**Supplementary Table 11.** Gene function annotation

| **Database** | **Count** | **Percentage** |
| --- | --- | --- |
| KO | 6609 | 31.693 |
| eggNOG | 9364 | 44.905 |
| GO | 11,774 | 56.462 |
| PFAM | 3921 | 18.803 |
| NR | 17,354 | 83.221 |
| Map | 10,683 | 51.23 |
| NT | 11,384 | 54.592 |
| Total_anno | 17,681 | 84.789 |
| Total_unigene | 20,853 | 100 |

**Supplementary Table 12.** Assembled genome assessment with BUSCOs

| **Item** | **Number** | **Percent(%)** |
| --- | --- | --- |
| Complete BUSCOs (C) | 1594 | 96.1 |
| Complete and single-copy BUSCOs (S) | 1483 | 89.4 |
| Complete and duplicated BUSCOs (D) | 111 | 6.7 |
| Fragmented BUSCOs (F) | 19 | 1.1 |
| Missing BUSCOs (M) | 45 | 2.8 |
| Total BUSCO groups searched | 1658 | - |


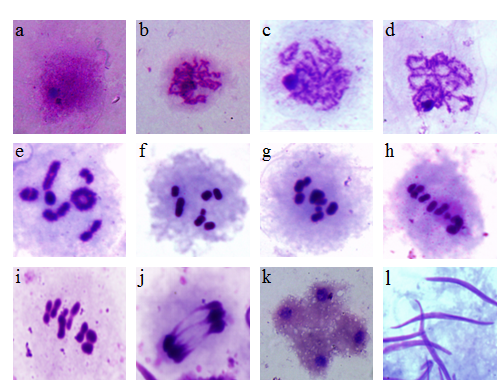


**Supplementary Figure 1.** Male meiotic chromosomes of *A. chinensis* after standard Feulgen–Giemsa staining. **a.** Leptotene, **b.** pachytene, **c.** diffuse, **d.** early diplotene, **e.** diplotene, **f.** diakinesis, **g.** metaphaseⅠ, **h.** anaphaseⅠ, **i.** metaphaseⅡ, **j.** telophaseⅡ, **k.** tetrad, **l.** sperm.


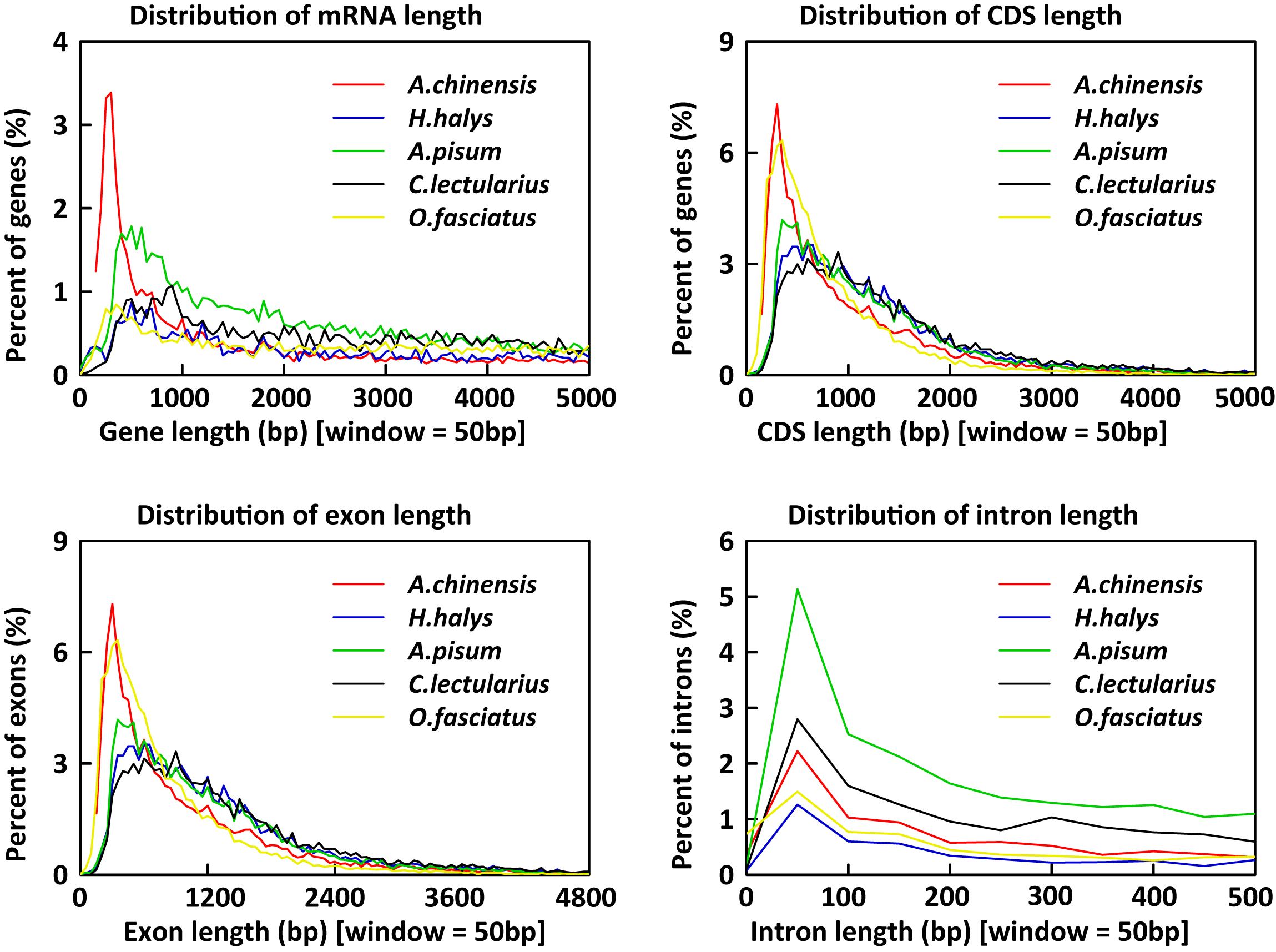


**Supplementary Figure 2**. Distribution of mRNA, coding sequence (CDS), exon, and intron lengths between *A. chinensis* and closely related species.


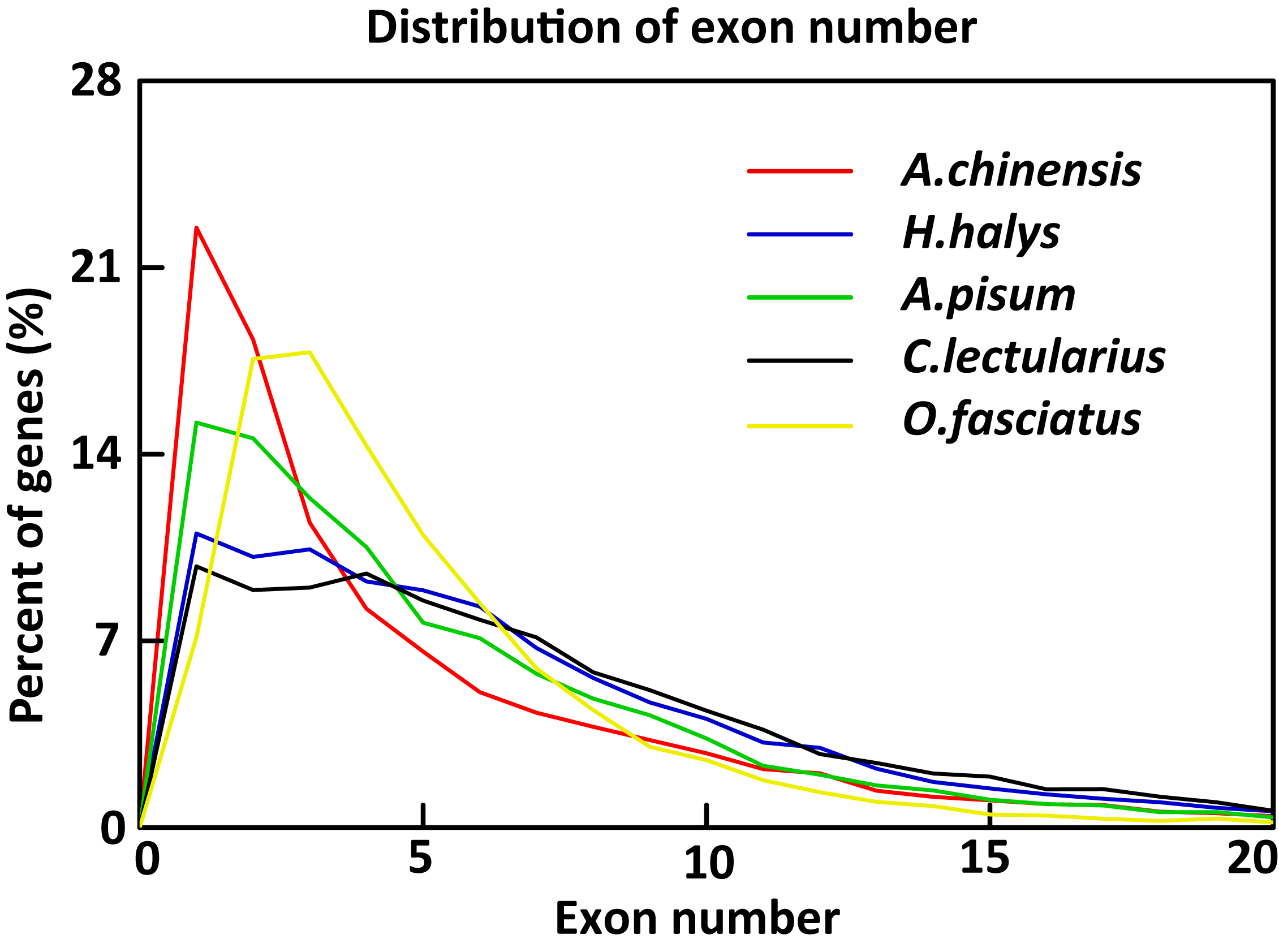


**Supplementary Figure 3.** Distribution of exon numbers between *A. chinensis* and closely related species.


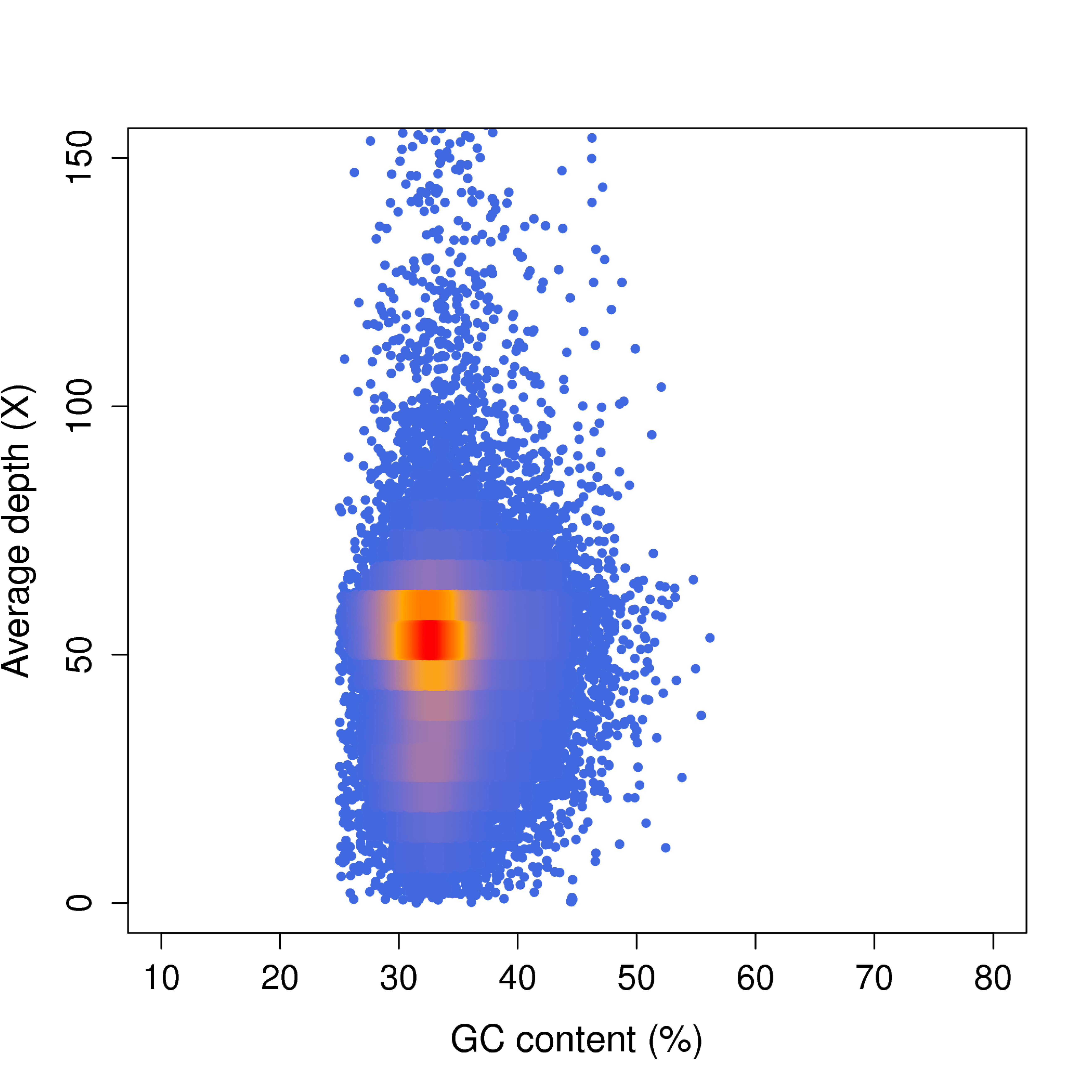


**Supplementary Figure 4.** GC depth scatter distribution.
